# High visceral adipose tissue area is independently associated with early allograft dysfunction in liver transplantation recipients: a propensity score analysis

**DOI:** 10.1186/s13244-022-01302-8

**Published:** 2022-10-11

**Authors:** Guanjie Yuan, Shichao Li, Ping Liang, Gen Chen, Yan Luo, Yaqi Shen, Xuemei Hu, Daoyu Hu, Jiali Li, Zhen Li

**Affiliations:** Department of Radiology, Tongji Hospital, Tongji Medical College, Huazhong University of Science and Technology, Wuhan, 430030 Hubei China

**Keywords:** Tomography (X-ray computed), Body composition, Liver transplantation, Allograft, Propensity score

## Abstract

**Objectives:**

To evaluate the association between adipose tissue distribution and early allograft dysfunction (EAD) in liver transplantation (LT) recipients.

**Methods:**

A total of 175 patients who received LT from April 2015 to September 2020 were enrolled in this retrospective study. The areas of abdominal adipose tissue and skeletal muscle of all patients were measured based on the preoperative CT images. The appropriate statistical methods including the propensity score-matched (PSM) analysis were performed to identify the association between adipose tissue distribution and EAD.

**Results:**

Of 175 LT recipients, 55 patients (31.4%) finally developed EAD. The multivariate logistic analysis revealed that preoperative serum albumin (odds ratio (OR) 0.34, 95% confidence interval (CI) 0.17–0.70), platelet–lymphocyte ratio (OR 2.35, 95% CI 1.18–4.79), and visceral adipose tissue (VAT) area (OR 3.17, 95% CI 1.56–6.43) were independent associated with EAD. After PSM analysis, VAT area was still significantly associated with EAD (OR 3.95, 95% CI 1.16–13.51). In survival analysis, no significant difference was identified in one-year graft failure (log-rank: *p *= 0.487), and conversely result was identified in overall survival (OS) (log-rank: *p* = 0.012; hazard ratio (HR) 4.10, 95% CI 1.27–13.16).

**Conclusions:**

LT recipients with high VAT area have higher risk for the occurrence of EAD, and high VAT area might have certain clinical value for predicting the poor OS of patients. For LT candidates with large amount of VAT, the clinicians can take clinical interventions by suggesting physical and nutritional treatments to improve outcomes after LT.

## Key points

- Liver transplantation (LT) recipients with high visceral adipose tissue (VAT) area were more likely to develop early allograft dysfunction.
- High VAT area may be associated with poor OS in LT recipients.
- LT candidates with high VAT area may be targets for timely therapeutic interventions.

## Introduction

Liver transplantation (LT) is the only effective therapeutic way for patients with end-stage liver disease [1]. In the past decades, several developments in surgical techniques, immunosuppression, and perioperative care have dramatically improved the postoperative outcomes [2, 3]. However, a considerable number of patients still develop graft insufficiency and failure after LT due to various kinds of postoperative complications, which may cause declines of survival rate and quality of life of patients.

EAD is a common and critical complication after LT, which indicates early graft function insufficiency and adversely influences patient and graft survival [4–6]. EAD was defined as the presence of at least one of the following criteria: total bilirubin ≥ 10 mg/dL on postoperative day 7; international normalized ratio (INR) ≥ 1.6 on postoperative day 7; and alanine aminotransferase (ALT) or aspartate aminotransferase (AST) > 2000 IU/L within the first postoperative 7 days [7]. Previous studies have shown that multiple factors lead to the occurrence of EAD, among which the recipient-related factors play an important role in the development of EAD [8–10]. The early prediction of EAD is helpful to discern recipients who may develop poor outcomes and stratify recipients for personalized treatment.

Obesity is known to be one of the important risk factors associated with poor post-transplant outcomes in terms of increased morbidity, which has long been considered a relative contraindication for organ transplantation [11–14]. Obesity was defined by the presence of excessive body fat accumulation to the extent that can lead to a variety of diseases and pathologies, including type 2 diabetes, hypertension, cardiovascular disease and nonalcoholic steatohepatitis, and several cancers [15]. The body mass index (BMI) has been considered as a standard of measuring obesity for a long time. Multiple previous studies have shown that BMI demonstrates association with outcomes after LT [16, 17]. However, recent reports have shown that the adipose tissue distribution measured by axial computed tomography (CT) imaging could distinguish between visceral and subcutaneous fat, which can provide more accurate and direct measurement of obesity, over and above the BMI. Many researches have revealed the fat distribution correlates significantly with complications and mortality in various kinds of disease [18–21]. To date, few studies have directly investigated measured the association between body fat distribution and EAD after LT. Thus, this retrospective study aims to determine the association between preoperative adipose tissue distribution and EAD in LT recipients and further explore the value of the most meaningful body distribution indicator for predicting overall survival (OS) and one-year graft failure of patients.

## Methods

### Study population

**Fig. 1:**
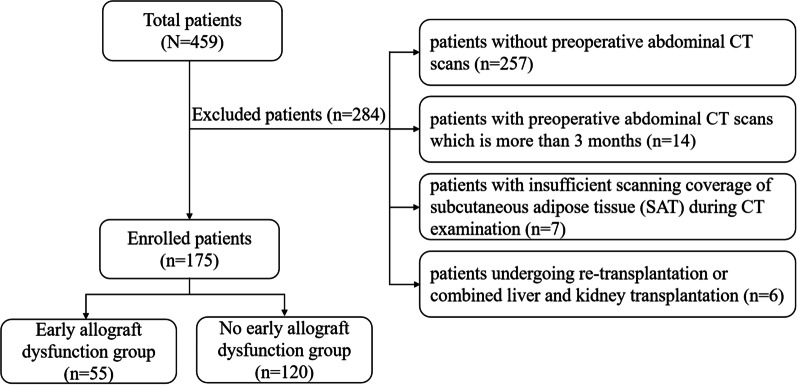
Flowchart of the study population

The Institutional Review Board of local hospital approved this study and the requirement for patient informed consent was waived. This study retrospectively evaluated 459 adult patients (age ≥ 18 years) who underwent LT from April 2015 to September 2020. 284 patients were excluded based on the following criteria: (1) patients without preoperative abdominal CT scans (*n* = 257); (2) patients with preoperative abdominal CT scans which is more than 3 months (*n* = 14); (3) patients with insufficient scanning coverage of subcutaneous adipose tissue (SAT) during CT examination (*n* = 7); and (4) patients who received re-transplantation or combined liver and kidney transplantation (*n* = 6). Finally, 175 patients were included in this study and their CT images were analyzed (Fig. 1). For patients with multiple abdominal CT examinations within 3 months before LT, CT scan with the shortest time interval between imaging and the operation was used for this analysis.

### Data collection

**Fig. 2:**
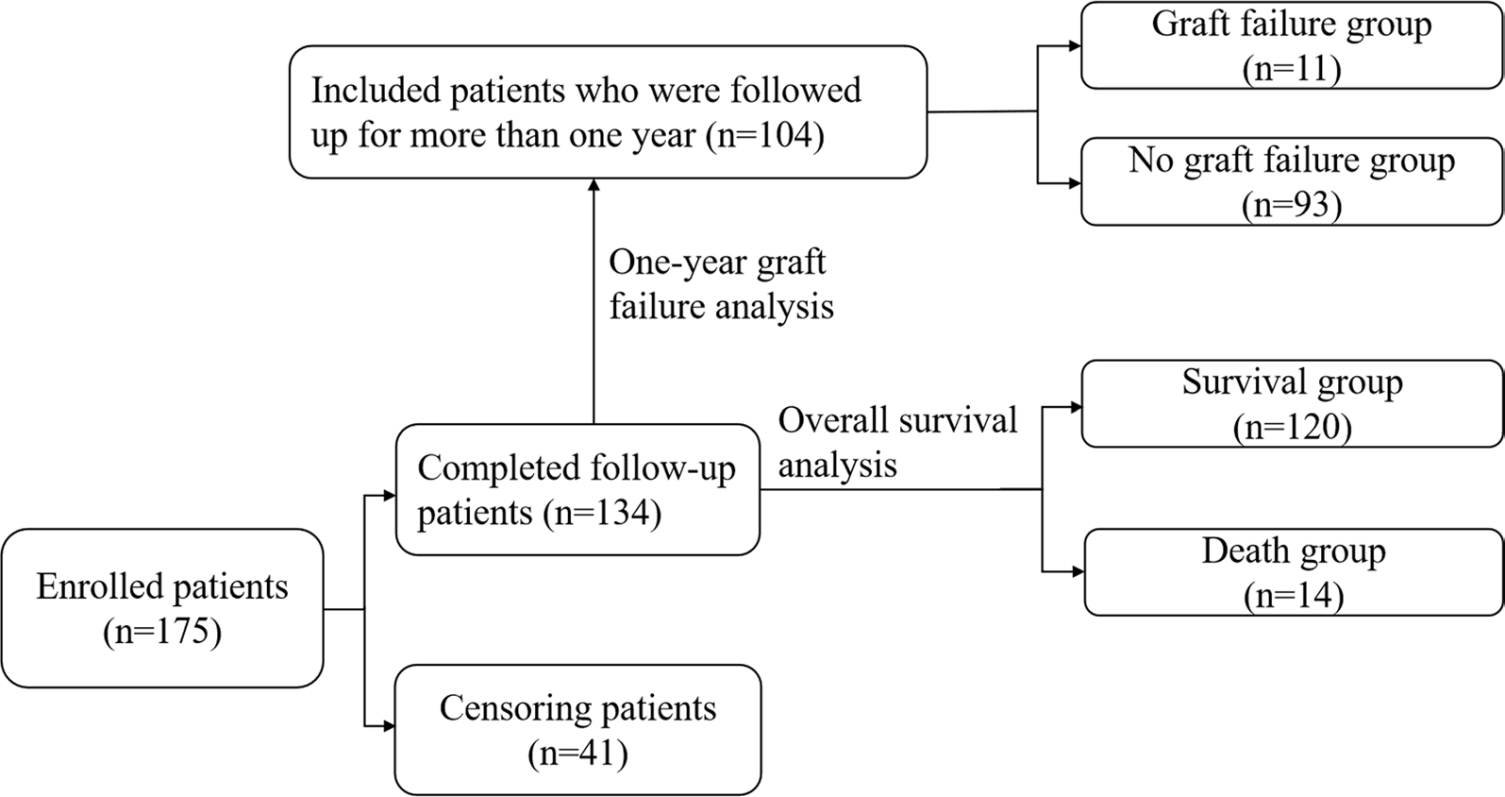
Flowchart of the follow-up

Clinical information of all patients, including baseline characteristics and perioperative laboratory variables were extracted from electronic medical records. BMI (weight in kg divided by height in meters squared) was based on weight and height measured during this hospital stay within one week prior to surgery. Patients were followed from the date of operation until April 10, 2021, or death. Follow-up time varied from 8 to 72 months, and the mean follow-up time was 28 months. Of 175 LT recipients, 134 patients were tracked until the end of follow-up. The patients who were tracked for less than one year form the date of operation to the end of follow-up were excluded in one-year graft survival analysis (*n* = 30) (Fig. 2).

### Outcome parameters

The primary outcome measurement for this study was EAD, which was defined as the presence of at least one of the following criteria: (1) bilirubin ≥ 10 mg/dL on postoperative day 7; (2) international normalized ratio (INR) ≥ 1.6 on postoperative day 7; (3) alanine aminotransferase (ALT) or aspartate aminotransferase (AST) > 2000 IU/L within the first postoperative 7 days [7]. The secondary outcomes were overall survival and one-year graft failure. One-year graft failure was defined as re-transplantation or death, whichever was first in one year after LT [22].

### CT protocol

All preoperative abdominal CT examinations were performed at our institution using CT scanners (Discovery CT 750 HD, GE Healthcare, USA; Somatom Force/Somatom Definition AS+, Siemens Healthineers, Germany; uCT 780, United Imaging, China) in a supine, feet‐first position. Intravenous contrast media 370 mg/mL iopromide (Ultravist 370, Bayer Schering Pharma, Berlin, Germany) was administered at a flow rate of 3.5 mL/s, followed by a 20-mL saline flush. The total contrast volume was 1.5 mL/kg. Contrast material was injected through the ante-cubital vein with an 18-gauge intravenous cannula using a dual-head injector (Stellant, Medrad, CO, USA), each with an injection time of 20 s.

The time of arterial phase scanning was determined when a threshold enhancement of 120 HU was achieved in the abdominal aorta. Portal phase imaging was initiated 25–30 s after the completion of arterial phase scanning. The main CT scanning parameters were as follows: tube voltage, 120 kV; automatic tube current modulation; scanning thickness, 10 mm; table speed, 39.37 mm/rotation; rotation time, 0.5 s; detector pitch, 0.984:1; matrix size = 512 × 512. All CT images were then reconstructed with a slice thickness of 5 mm.

### CT measurements

**Fig. 3:**
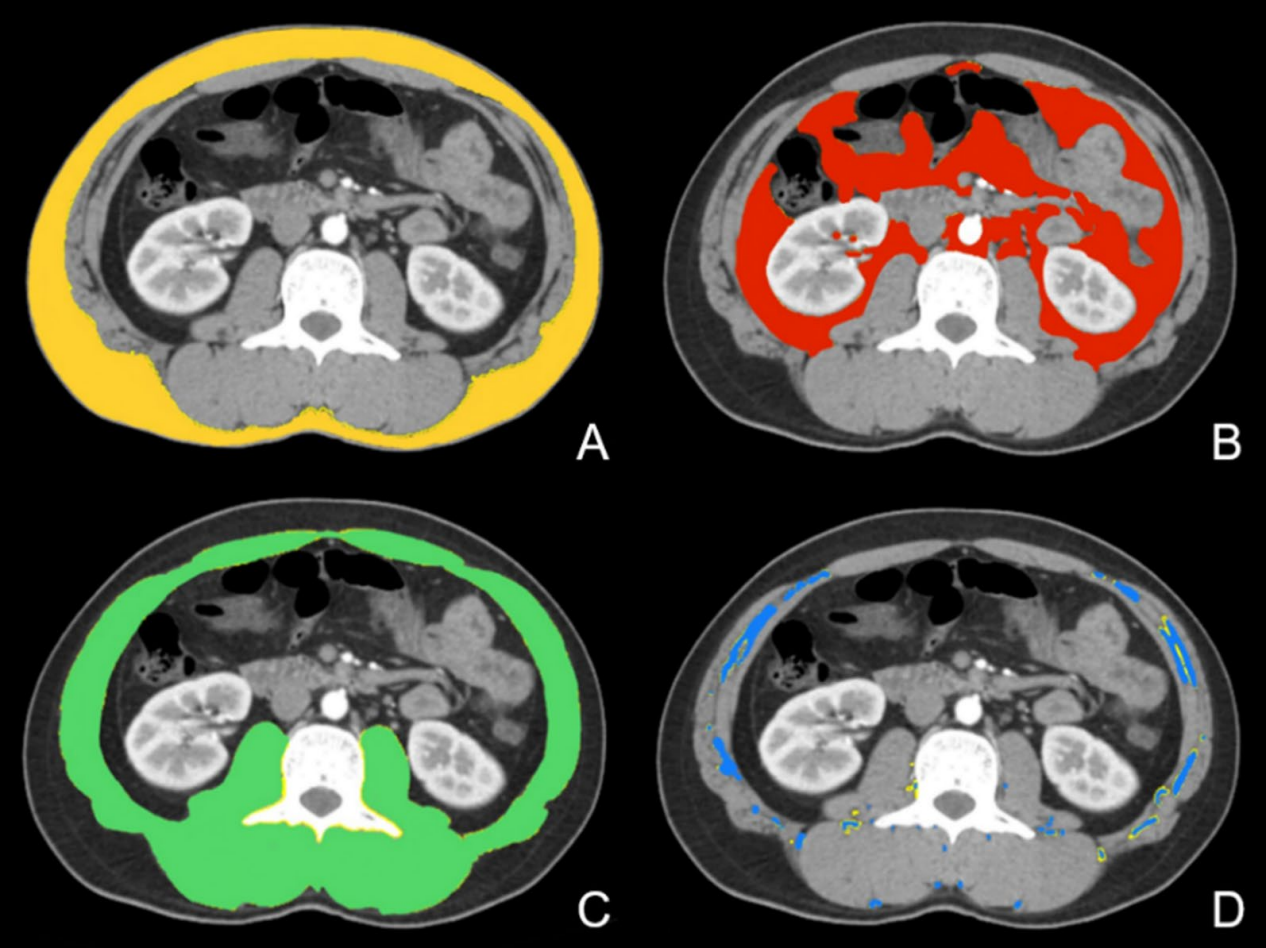
Cross‐sectional computed tomography images at the level of the third lumbar vertebra. **A** Subcutaneous adipose tissue area (yellow) was quantified using − 190 to − 30 Hounsfield units (HU). **B** Visceral adipose tissue area (red) was quantified using − 150 to − 50 HU. **C** Skeletal muscle area (green) was quantified using − 29 to 150 HU. **D** Intramuscular adipose tissue area (blue) was quantified using − 190 to − 30 HU

The cross-sectional CT image at the level of third lumbar vertebra (L3) to quantify skeletal muscle and abdominal adipose tissue area was analyzed [23]. Preoperative CT images were analyzed using ImageJ software (http://rsbweb.nih.gov/ij/download.html). A semi-automated method defined by density window was used to measure the body composition variables by manually outlining of the border of the muscle and excluding of the bowel contents. Skeletal muscles include the psoas, erector spinae, quadratus lumborum, transversus abdominis, latissimus dorsi, external and internal obliques, and rectus abdominis [24]. Tissue Hounsfield unit (HU) thresholds were employed as follows: − 29 HU to 150 HU for skeletal muscle area (SMA), − 190 HU to − 30 HU for SAT area and intramuscular adipose tissue (IMAT) area, and − 150 HU to − 50 HU for visceral adipose tissue (VAT) area [25, 26]. The different portions of adipose tissue distribution at L3 are shown in Fig. 3. The mean attenuation of the entire skeletal muscle (SMD) was also measured. The increase in IMAT and correspondingly decline of SMD represent decrease in strength and muscle quality [26]. The visceral to subcutaneous adipose tissue area ratio (VSR) was also calculated, and it was usually used to reflect visceral adiposity [19].

### Statistical analysis

Continuous variables were presented as median (interquartile range, IQR) and were compared by an independent sample Student t test or Mann–Whitney U test. Categorical variables were presented as number (percentage) and compared by the Chi-squared analysis or Fisher’s exact test. Variables with *p* ≤ 0.10 in univariate analysis were considered candidates for multivariate logistic regression analysis to identify the risk factors associated with EAD. To further demonstrate the relationship between VAT area and EAD, one-to-one propensity-score matching (PSM) method (*n* = 45 pairs) was used to minimize the impact of selection bias and potential confounding factors between different groups. The following variables were entered into the propensity model: age, gender, BMI, serum albumin, platelet–lymphocyte ratio (PLR). The propensity scores for subjects with and without EAD were matched within a caliper of 0.02. The ideal cut-of value of was set as the value maximizing the Youden index according to the receiver operating characteristic (ROC) curve. Cumulative rates of overall survival and one-year graft failure were calculated using Kaplan–Meier method and log-rank test. The hazard ratio (HR) was obtained by Cox univariate regression analysis. Statistical analysis was performed with SPSS version 25 statistical software (Chicago, IL, USA) and MedCalc (https://www.medcalc.org/). All tests were two-sided, and values of *p* ≤ 0.05 were considered statistically significant.

## Results

### Patient characteristics

Out of all 459 LT cases performed within the given time frame, a total of 175 recipients were enrolled in this study and EAD occurred in 55 (31.4%) cases. The clinical and body composition characteristics of patients in the two groups are summarized in Table 1. Median recipient age at transplant was 49 (42–54) [IQR] years, and 81.1% (*n* = 142) of patients were male. No significant differences in age and gender were found between the two groups (*p* = 0.435; *p* = 0.069, respectively). The median BMI value was 22.5 (20.3–24.7) [IQR] kg/m^2^ in all patients, and the EAD group was significantly more likely to have higher BMI value than No EAD group (23.2 vs 22.0, *p* = 0.019). The most common reasons for LT were hepatocellular carcinoma (45.7%), viral hepatitis infection (38.3%), followed by primary biliary cirrhosis and autoimmune liver disease (8.0%), other diseases (4.6%), and alcoholic cirrhosis (3.4%). The hepatic features including Child–Pugh score and MELD score were slightly higher in the EAD group than in the No EAD group, but the difference was not statistically significant (7 vs 6, *p* = 0.189; 13 vs 12, *p* = 0.507, respectively).

**Table 1:** Clinical and body composition characteristics of the patients

Variables	Total (*n* = 175)	EAD (*n* = 55)	No EAD (*n* = 120)	*p* value
Age (years), median (IQR)	49 (42–54)	43 (50–55)	49 (41–54)	0.435
Gender (M/F), *N* (%)	142/33 (81.1/18.9)	49/6 (89.1/10.9)	93/27 (77.5/22.5)	0.069
**BMI (kg/m**^**2**^**), median (IQR)**	**22.5 (20.3–24.7)**	**23.2 (20.8–24.9)**	**22.0 (19.9–24.2)**	**0.019**
Viral status, *N* (%)				0.316
HBV/HCV/HBV + HCV/None	132/8/1/34 (75.4/4.6/0.6/19.4)	42/1/1/11(76.4/1.8/1.8/20.0)	90/7/0/23 (75.0/5.8/0.0/19.2)	
Etiology, *N* (%)				0.377
Viral hepatitis	67 (38.3%)	20 (36.4%)	47 (39.2%)	
Alcoholic cirrhosis	6 (3.4%)	2 (3.6%)	4 (3.3%)	
Hepatocellular carcinoma	80 (45.7%)	29 (52.7%)	51 (42.5%)	
Primary biliary cirrhosis and Autoimmune liver disease	14 (8.0%)	2 (3.6%)	12 (10.0%)	
Other	8 (4.6%)	2 (3.6%)	6 (10.9%)	
Coexisting conditions, *N* (%)				
Smoking	70 (40.0%)	22 (40.0%)	48 (40.0%)	> 0.999
Drinking	54 (30.9%)	18 (32.7%)	36 (30.0%)	0.717
Hypertension	15 (8.6%)	6 (10.9%)	9 (7.5%)	0.455
Diabetes	18 (10.3%)	4 (7.3%)	14 (11.7%)	0.434
Cardiovascular and cerebrovascular disease	8 (4.6%)	3 (5.5%)	5 (4.2%)	0.709
Chronic kidney disease	2 (1.1%)	1 (1.8%)	1 (0.8%)	0.531
Hepatic features				
Child–Pugh class A/B/C, *N* (%)	88/70/17 (50.3/40.0/9.7)	26/20/9 (47.3/36.4/16.4)	62/50/8 (51.7/41.7/6.7)	0.131
Child–Pugh score, median (IQR)	6 (5–8)	7 (5–9)	6 (5–8)	0.189
MELD Score, median (IQR)	12 (9–18)	13 (9–18)	12 (8–17)	0.507
Preoperative laboratory values				
ALT (U/L), median (IQR)	29 (19–47)	31 (20–54)	29 (18–45)	0.423
AST (U/L), median (IQR)	39 (28–72)	44 (27–77)	39 (28–64)	0.522
ALP (U/L), median (IQR)	104 (79–155)	106 (83–144)	103 (76–163)	0.778
LDH (U/L), median (IQR)	209 (171–267)	207 (173–271)	209 (166–261)	0.714
Total bilirubin (μmol/L), median (IQR)	30.6 (16.7–79.2)	31.6 (18.4–99.7)	27.4 (14.9–73.1)	0.411
Albumin (g/L), median (IQR)	36.2 (31.9–40.8)	35.4 (31.4–39.2)	37.1 (32.6–41.4)	0.085
Creatinine (μmol/L), median (IQR)	68 (57–76)	69 (61–75)	66 (56–76)	0.584
Blood ammonia(μmol/L), median (IQR)	57 (44–74)	60 (49–79)	55 (43–74)	0.299
Neutrophil (× 10^9^/L), median (IQR)	2.58 (1.53–4.57)	2.61 (1.35–5.37)	2.47 (1.59–3.94)	0.908
Lymphocyte (× 10^9^/L), median (IQR)	0.95 (0.56–1.39)	1.00 (0.51–1.42)	0.89 (0.57–1.36)	0.742
Platelet (× 10^9^/L), median (IQR)	72 (44–131)	84 (51–139)	63 (42–128)	0.113
NLR, median (IQR)	2.71 (1.90–4.98)	2.50 (1.64–5.80)	2.80 (1.92–4.86)	0.726
PLR, median (IQR)	86.67 (56.53–138.52)	104.43 (65.77–170.0)	84.61 (51.68–132.48)	0.100
Prothrombin time(s), median (IQR)	16.6 (14.6–19.6)	16.6 (14.5–20.1)	16.6 (14.6–19.5)	0.890
INR, median (IQR)	1.35 (1.14–1.65)	1.36 (1.13–1.72)	1.35 (1.14–1.64)	0.900
Body composition variable				
SMA (cm^2^), median (IQR)	137.3 (113.2–156.4)	143.4 (123.0–166.2)	132.2 (109.4–156.2)	0.075
SMD (HU), median (IQR)	37.3 (43.6–47.9)	43.0 (34.2–48.6)	44.0 (38.7–47.7)	0.350
**SAT (cm**^**2**^**), median (IQR)**	**90.6 (61.5–131.5)**	**109.3 (76.0–149.2)**	**84.6 (49.5–128.4)**	**0.013**
**VAT (cm**^**2**^**), median (IQR)**	**77.9 (49.3–120.7)**	**101.0 (61.3–157.0)**	**69.6 (39.1–108.0)**	**< 0.001**
VSR, median (IQR)	0.87 (0.64–1.14)	0.95 (0.71–1.22)	0.86 (0.60–1.08)	0.084
IMAT (cm^2^), median (IQR)	3.1 (2.0–5.4)	3.4 (2.4–6.0)	2.9 (1.9–5.2)	0.068

The bold indicated the items with statistically significant difference between the two groups

EAD, early allograft dysfunction; BMI, body mass index; HBV, hepatitis B virus; HCV, hepatitis C virus; MELD, model for end‐stage liver disease; ALT, alanine aminotransferase; AST, aspartate aminotransferase; ALP, alkaline phosphatase; LDH, lactate dehydrogenase; NLR, Neutrophil–lymphocyte ratio; PLR, platelet–lymphocyte ratio; INR, international normalized ratio; SMA, skeletal muscle area; SMD, the mean attenuation of skeletal muscle; SAT, subcutaneous adipose tissue; VAT, visceral adipose tissue; VSR, visceral to subcutaneous adipose tissue area ratio; IMAT, intramuscular adipose tissue

### Body composition analysis

The median SAT and VAT area were 90.6 (61.5–131.5) [IQR] cm^2^ and 77.9 (49.3–120.7) [IQR] cm^2^ in all patients, respectively. Significant differences were observed between EAD group and No EAD group in SAT and VAT area (109.3 vs 84.6, *p* = 0.013; 101.0 vs 69.6, *p* < 0.001, respectively). There were no significant differences between EAD group and No EAD group in SMA and VSR (143.4 vs 132.2, *p* = 0.075; 0.95 vs 0.86, *p* = 0.084, respectively). The EAD group has higher IMAT area and correspondingly lower SMD value, but the differences were not statistically significant (3.4 vs 2.9, *p* = 0.068; 43.0 vs 44.0, *p* = 0.350, respectively).

### Multivariate logistic analysis of risk factors for EAD

Nine of the examined variables with *p* value close to 0.10 in univariate analysis including gender, BMI, preoperative serum albumin, PLR, SMA, SAT, VAT, VSR, and IMAT were applied to multivariate analysis to identify the risk factors associated with EAD (*p* = 0.069; *p* = 0.019; *p* = 0.085; *p* = 0.100; *p* = 0.075; *p* = 0.013; *p* < 0.001; *p* = 0.084; *p* = 0.068, respectively) (Table 2). However, on multivariate analysis, only preoperative serum albumin (odds ratio (OR) 0.34, 95% confidence interval (CI) 0.17–0.70, *p* = 0.004), PLR (OR 2.35, 95% CI 1.18–4.79, *p* = 0.018), VAT area were independently associated with EAD and the VAT area was with the biggest OR among the three variables (OR 3.17, 95% CI 1.56–6.43, *p* = 0.001).

**Table 2:** Multivariate analysis of risk factors for early allograft dysfunction (EAD)

Variables	EAD (*n* = 55)	No EAD (*n* = 120)	*p* value (≤ 0.10)	Multivariate analysis		
				OR	95% CI	*p* value
Gender (M/F), *N* (%)	49/6 (89.1/10.9)	93/27 (77.5/22.5)	0.069			
BMI (kg/m^2^), median (IQR)	23.2 (20.8–24.9)	22.0 (19.9–24.2)	0.019			
**Albumin (g/L), median (IQR)**	**35.4 (31.4–39.2)**	**37.1 (32.6–41.4)**	**0.085**	**0.34**	**0.17–0.70**	**0.004**
**PLR, median (IQR)**	**104.43 (65.77–170.0)**	**84.61 (51.68–132.48)**	**0.100**	**2.35**	**1.18–4.79**	**0.018**
SMA (cm^2^), median (IQR)	143.4 (123.0–166.2)	132.2 (109.4–156.2)	0.075			
SAT (cm^2^), median (IQR)	109.3 (76.0–149.2)	84.6 (49.5–128.4)	0.013			
**VAT (cm**^**2**^**), median (IQR)**	**101.0 (61.3–157.0)**	**69.6 (39.1–108.0)**	** < 0.001**	**3.17**	**1.56–6.43**	**0.001**
VSR, median (IQR)	0.95 (0.71–1.22)	0.86 (0.60–1.08)	0.084			
IMAT (cm^2^), median (IQR)	3.4 (2.4–6.0)	2.9 (1.9–5.2)	0.068			

The bold indicated the items with statistically significant difference

EAD, early allograft dysfunction; OR, odds ratio; 95% CI, 95% confidence interval; BMI, body mass index; PLR, platelet–lymphocyte ratio; SMA, skeletal muscle area; SAT, subcutaneous adipose tissue; VAT, visceral adipose tissue; VSR, visceral to subcutaneous adipose tissue area ratio; IMAT, intramuscular adipose tissue

### Propensity score-matched analysis

PSM analysis was performed to minimize the potential bias and further verify the accuracy of VAT area as risk factor of EAD. Patients were matched for age, gender, BMI, serum albumin, and PLR. Finally, 45 pairs of patients were selected using one-to-one PSM method (Table 3). Five of examined variables with *p* value close to 0.10 in univariate analysis including Child–Pugh class, SMD, VAT, VSR, and IMAT were applied to multivariate analysis (*p* = 0.085; *p* = 0.059; *p* = 0.047; *p* = 0.042; *p* = 0.042, respectively), and VAT area was still independently associated with EAD in multivariate analysis (OR 3.95, 95% CI 1.16–13.51, *p* = 0.029) (Table 4). Besides, SMD exhibited significant association with EAD in multivariate analysis after PSM (OR 3.84, 95% CI 1.23–12.00, *p* = 0.020).

**Table 3:** Clinical and body composition characteristics of patients after propensity scoring-matched (PSM) analysis

Variables	Total (*n* = 90)	EAD (*n* = 45)	No EAD (*n* = 45)	*p* value
Age (years), median (IQR)	50 (42–54)	50 (41–55)	48 (42–53)	0.324
Gender (M/F), *N* (%)	81/9 (90.0/10.0)	39/6 (86.7/13.3)	42/3 (93.3/6.7)	0.482
BMI (kg/m^2^), median (IQR)	22.9 (20.7–24.8)	22.9 (20.8–24.7)	22.7 (20.7–25.5)	0.993
Viral status, *N* (%)				0.221
HBV/HCV/HBV + HCV/none	72/3/1/14 (80.0/3.3/1.1/15.6)	33/1/1/10 (73.3/2.2/2.2/22.2)	39/2/0/4 (86.7/4.4/0.0/8.9)	
Etiology, *N* (%)				0.780
Viral hepatitis	33 (36.7%)	16 (35.6%)	17 (37.8%)	
Alcoholic cirrhosis	4 (4.4%)	2 (4.4%)	2 (4.4%)	
Hepatocellular carcinoma	49 (54.4%)	24 (53.3%)	25 (55.6%)	
Primary biliary cirrhosis & Autoimmune liver disease	3 (3.3%)	2 (4.4%)	1 (2.2%)	
Other	1 (1.1%)	1 (2.2%)	0 (0.0%)	
Coexisting conditions, *N* (%)				
Smoking	39 (43.3%)	17 (37.8%)	22 (48.9%)	0.288
Drinking	27 (30.0%)	12 (26.7%)	15 (33.3%)	0.490
Hypertension	5 (11.1%)	4 (8.9%)	1 (2.2%)	0.357
Diabetes	8 (8.9%)	4 (8.9%)	4 (8.9%)	> 0.999
Cardiovascular and cerebrovascular disease	3 (3.3%)	2 (4.4%)	1 (2.2%)	> 0.999
Chronic kidney disease	2 (2.2%)	1 (2.2%)	1 (2.2%)	> 0.999
Hepatic features				
Child–Pugh class A/B/C, *N* (%)	45/36/9 (50.0/40.0/10.0)	24/14/7 (53.3/31.1/15.6)	21/22/2 (46.7/48.9/4.4)	0.085
Child–Pugh score, median (IQR)	7 (5–8)	6 (5–9)	7 (5–8)	0.798
MELD Score, median (IQR)	12 (9–16)	12 (9–17)	13 (8–16)	0.987
Preoperative laboratory values				
ALT (U/L), median (IQR)	29 (19–51)	27 (19–53)	30 (19–49)	0.824
AST (U/L), median (IQR)	40 (28–71)	40 (27–73)	39 (29–62)	0.929
ALP (U/L), median (IQR)	105 (82–140)	104 (84–141)	105 (78–139)	0.837
LDH (U/L), median (IQR)	201 (174–270)	204 (177–270)	198 (173–269)	0.865
Total bilirubin (μmol/L), median (IQR)	31.0 (16.6–74.8)	29.7 (17.9–82.2)	36.5 (12.4–76.5)	0.862
Albumin (g/L), median (IQR)	35.8 (31.4–40.2)	35.8 (31.7–40.7)	35.8 (31.1–40.2)	0.842
Creatinine (μmol/L), median (IQR)	68 (59–75)	69 (60–73)	66 (58–79)	0.762
Blood Ammonia(μmol/L), median (IQR)	56 (46–73)	57 (48–63)	56 (41–75)	0.900
Neutrophil (× 10^9^/L), median (IQR)	2.44 (1.52–4.27)	2.51 (1.31–4.57)	2.39 (1.67–4.09)	0.594
Lymphocyte (× 10^9^/L), median (IQR)	1.09 (0.63–1.49)	1.10 (0.64–1.55)	1.09 (0.61–1.47)	0.815
Platelet (× 10^9^/L), median (IQR)	76 (50–162)	84 (51–132)	68 (49–174)	0.981
NLR, median (IQR)	2.60 (1.71–4.82)	2.33 (1.60–4.77)	2.75 (1.83–4.92)	0.490
PLR, median (IQR)	82.61 (60.68–143.02)	78.26 (60.87–139.49)	90.00 (56.44–145.11)	0.821
Prothrombin time(s), median (IQR)	16.5 (14.5–19.1)	16.4 (14.2–20.2)	16.6 (14.6–18.7)	0.834
INR, median (IQR)	1.33 (1.13–1.62)	1.32 (1.12–1.73)	1.35 (1.14–1.54)	0.929
Body composition variable				
SMA (cm^2^), median (IQR)	143.4 (127.6–162.2)	143.4 (123.5–158.8)	142.0 (127.7–164.4)	0.588
SMD (HU), median (IQR)	45.5 (39.6–50.1)	44.4 (36.6–49.7)	45.8 (41.4–50.7)	0.059
SAT (cm^2^), median (IQR)	98.9 (69.3–130.1)	107.4 (73.6–135.3)	90.6 (57.7–131.8)	0.503
**VAT (cm**^**2**^**), median (IQR)**	**84.6 (51.8–127.4)**	**88.2 (59.1–141.8)**	**73.0 (30.1–122.7)**	**0.047**
**VSR, median (IQR)**	**0.86 (0.60–1.08)**	**0.93 (0.70–1.21)**	**0.80 (0.57–0.99)**	**0.042**
**IMAT (cm**^**2**^**), median (IQR)**	**3.0 (2.0–4.7)**	**3.3 (2.4–5.8)**	**2.5 (1.8–4.2)**	**0.042**

The bold indicated the items with statistically significant difference between the two groups

EAD, early allograft dysfunction; BMI, body mass index; HBV, hepatitis B virus; HCV, hepatitis C virus; MELD, model for end‐stage liver disease; ALT, alanine aminotransferase; AST, aspartate aminotransferase; ALP, alkaline phosphatase; LDH, lactate dehydrogenase; NLR, neutrophil–lymphocyte ratio; PLR, platelet–lymphocyte ratio; INR, international normalized ratio; SMA, skeletal muscle area; SMD, the mean attenuation of skeletal muscle; SAT, subcutaneous adipose tissue; VAT, visceral adipose tissue; VSR, visceral to subcutaneous adipose tissue area ratio; IMAT, intramuscular adipose tissue

**Table 4:** Multivariate analysis of risk factors for early allograft dysfunction after propensity scoring-matched (PSM) analysis

Variables	EAD (*n* = 45)	No EAD (*n* = 45)	*P* value (≤ 0.10)	Multivariate analysis		
				OR	95% CI	*p* value
Child–Pugh class A/B/C (%), *N* (%)	24/14/7 (53.3/31.1/15.6)	21/22/2 (46.7/48.9/4.4)	0.085			
**SMD (HU), median (IQR)**	**44.4 (36.6–49.7)**	**45.8 (41.4–50.7)**	**0.059**	**3.84**	**1.23–12.00**	**0.020**
**VAT (cm**^**2**^**), median (IQR)**	**88.2 (59.1–141.8)**	**73.0 (30.1–122.7)**	**0.047**	**3.95**	**1.16–13.51**	**0.029**
VSR, median (IQR)	0.93 (0.70–1.21)	0.80 (0.57–0.99)	0.042			
IMAT (cm^2^), median (IQR)	3.3 (2.4–5.8)	2.5 (1.8–4.2)	0.042			

The bold indicated the items with statistically significant difference

EAD, early allograft dysfunction; OR, odds ratio; 95% CI, 95% confidence interval; SMD, the mean attenuation of skeletal muscle; VAT, visceral adipose tissue; VSR, visceral to subcutaneous adipose tissue area ratio; IMAT, intramuscular adipose tissue

### Survival analysis

**Fig. 4:**
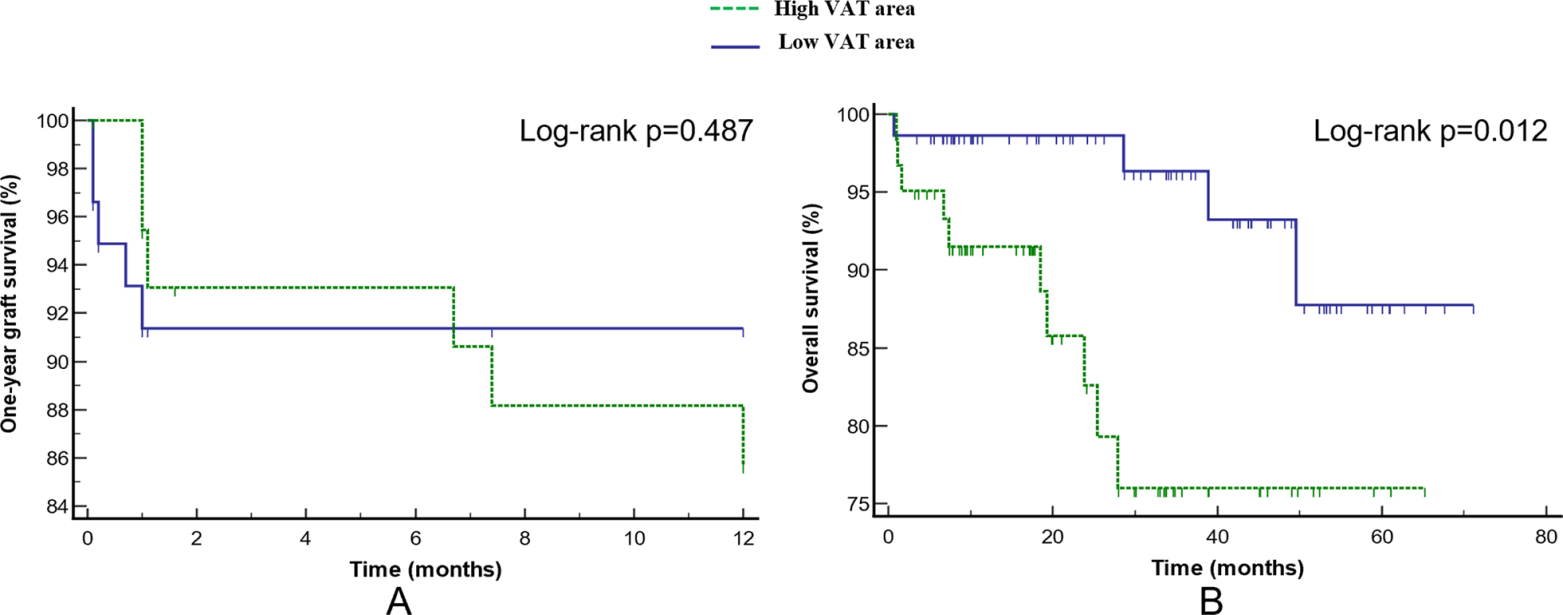
Kaplan–Meier curves of one-year graft failure and overall survival (OS) in high and low visceral adipose tissue (VAT) area group. **A** Kaplan–Meier curves of one-year graft failure in patients with high and low VAT area. **B** Kaplan–Meier curves of OS in patients with high and low VAT area

The VAT area of 85.2 cm^2^ was the optimal cut-off value with the highest Youden’s index (Youden’s index = 0.25, sensitivity = 64%, specificity = 62%) for predicting postoperative EAD. In one-year graft failure analysis, 45 patients were classified into high VAT area group among which 6 patients were confirmed graft failure and 59 patients were classified into low VAT area group among which 5 patients were confirmed graft failure. Kaplan–Meier analysis revealed that no significant difference was identified in one-year graft failure between the two groups (average survival time: 11.0 vs 10.9, *p* = 0.487) (Fig. 4a). In OS analysis, 61 patients were classified into high VAT area group among which 51 patients were alive until the end of the follow-up and 73 patients were classified into low VAT area group among which 69 patients were alive until the end of the follow-up. Kaplan–Meier analysis revealed that the difference was significantly between the two groups (average survival time 67.0 vs 53.5, *p* = 0.012) (Fig. 4b). The risk of death in the high VAT area group was 4.10 times that of the low VAT area group (HR 4.10, 95% CI 1.27–13.16, *p* = 0.018).

## Discussion

EAD is a critical complication after LT, which contributes to high mortality. Therefore, a better understanding of the risk factors associated with EAD can help to improve pre-LT patient management and post-LT outcome. In this study, we comprehensively analyzed the impact of adipose tissue distribution on outcomes in patients who underwent LT and determined that VAT area was independently associated with the development of EAD. In addition, we found that recipients with high visceral fat area have worse OS compared with those with relatively low visceral fat area. Therefore, the early identification of those patients who have excess visceral fat may not only prompt a therapeutic intervention, but also warn of an increased risk of poor outcomes.

### Limited value of BMI

Our current results indicated that 31.4% of the recipients occurred EAD after LT, which was consistent with the range of 5.2–38.7% reported in previous studies [4–8, 27]. In this study, EAD group tend to have slightly higher BMI value than No EAD group before PSM analysis. At present, the relationship between BMI and prognostic outcome post-transplant needs further verification. Although some studies have revealed an association between pretransplant high BMI value of recipients and poor survival after LT for both adult and pediatric recipients [15, 28, 29], recent studies reported that BMI was not associated with higher risk of post-transplant vascular and biliary complications, graft loss, and death [16, 30–32]. The inconsistent results could be attributed to different BMI grouping criteria and the limitations of BMI value in predicting outcomes after LT including the overestimated influence of fluid accumulation or systemic edema and inability to discriminate different components of body composition. Therefore, the application of BMI to reflect obesity and assess prognosis is limited. Body composition measurement based on CT images can provide more accurate information and may be regarded as a useful tool with prognostic value in recipients after LT.

### Potential fat distribution indices

Many recent reports have indicated that excessive accumulation of abdominal adipose tissue significantly correlates with postoperative outcomes, including complications and mortality in patients with various cancers [19, 20, 33, 34]. In this study, we observed significant differences between the two groups in the SAT and VAT area before PSM analysis. However, the difference was no longer significant in the SAT area after PSM analysis. The different results between VAT and SAT were on the basis of their differences in anatomical location, cellular, molecular, and metabolic activity [35]. Our results also found that the EAD group have significantly higher IMAT area than No EAD group after PSM analysis and the SMD exhibited significant association with EAD in multivariate analysis after PSM analysis. However, the SMA between two groups was not significantly different before and after PSM analysis. Although many previous studies have reported that low muscle mass was significantly associated with survival in patients who suffered from hepatocellular carcinoma or received LT [36–38], recent studies tend to show that muscle quality rather than muscle quantity was identified as a prognostic marker in LT recipients [24, 39]. IMAT is thought to begin to increase when lipids intake exceeds the disposal capacity of adipose tissue and muscle, and the increase represents the decline in muscle strength and quality [40]. The accumulation of IMAT may be associated with a muscle-to-liver cross-talking that the secretion of pro-inflammatory cytokines would increase and concentrations of myokines would decrease, which may in turn lead to systemic inflammation with unfavorable immune response and restricted graft regeneration [41]. In the study of Czigany et al. [24], the researchers have reported that patients with high IMAT accumulation and correspondingly low SMD, rather than reduced muscle mass, had significantly higher post-transplant complication rates and poor perioperative outcomes, which was similar with our results.

### Meaningful clinical parameters

In this study, multivariate analysis showed that serum albumin, PLR, VAT area were significantly associated with EAD before PSM analysis. The relationship between serum albumin and post-transplant outcomes remains controversial. Many previous studies have demonstrated that there is no significant correlation between preoperative serum albumin and patient or graft survival [42, 43]. However, in the studies of Hiroi et al. [44] and Bernardi et al. [45], serum albumin can influence short-term outcomes following LT and the albumin administration to patients on wait-listed for LT should be strengthen, which was in line with our results. They hold the view that maintaining high serum albumin level reflects good nutritional status and can reduce the amount of fluid collection in abdominal or thoracic cavity, which could resist the catabolic state induced by surgical stress and inflammatory response in the early postoperative period. As for PLR, our findings stay consistent with several observations of the PLR prognostic role in patients undergoing LT [46, 47]. Pravisani et al. [47] reported that pre-LT and post-LT PLR has shown clear associations with short- or long-term outcomes and HCC recurrence, which can be used as inflammatory and nutritional biomarkers to offer reliable prognostic information after LT. Elevated PLR reflects the more severer liver inflammation and worse nutritional status, and this may be the reason why PLR was significantly associated with EAD [48].

### Remarkable performance of VAT

Previous studies have revealed that high VAT area measured by CT is associated with greater risk of post-transplant complications and outcomes. For instance, in the study of Kamo et al. [49], the authors found that incidence of post-transplant bacteremia was significantly higher in patients with high visceral fat area. Terjimanian et al. [50] reported that excessive visceral fat was associated with a shorter one-year and five-year survival after LT. According to Montano-Loza et al. [51], increased visceral fat area was significantly associated with post-transplant tumor recurrence on 78 hepatocellular carcinoma liver transplant recipients. Our results showed that visceral, instead of the subcutaneous adipose deposition, worked as a significant risk factor for the development of EAD. After adjustment for potential confounders with PSM analysis, the independent association between VAT area and EAD still exists. In addition, our study also found that VAT area might also have certain clinical value for predicting the OS of recipients.

Prior researches have shown that VAT compared with SAT contains a larger number of inflammatory and immune cells. The cells will release more pro‐inflammatory cytokines such as tumor necrosis factor (TNF)-ɑ, interleukin (IL)-6, IL-1b and monocyte chemoattractant protein-1 to create a pro‐inflammatory microenvironment that potentially impairs immune function [52]. However, anti-inflammatory cytokines such as adiponectin is more highly secreted form SAT [53]. Thus, patients with high amount of adipose tissue in the visceral region are more easily to be in a state of chronic inflammation status. On the other hand, the pro‐inflammatory cytokines especially TNF-ɑ released by VAT plays an important role in the hepatic ischemia/reperfusion injury (IRI), which is considered to serve as pivotal mechanisms of influencing early and long-term results of the organ transplantation [54, 55]. Therefore, high VAT area can upregulate the release of pro‐inflammatory cytokines that contribute to the IRI, thereby promoting a higher incidence rate of EAD and having a negative impact on the long-term outcomes. In addition, it has been believed that VAT exerts damaging metabolic effects. Excessive accumulation of VAT has high rate of insulin resistance by provoking greater toxic-free fatty acids (FFA) release [56]. FFAs and adipokines secreted from VAT can flow into the liver through the portal vein and directly mediate the metabolic changes and injury of the graft [57]. As reported in previous researches, the accumulation of VAT contributes to increased risk of metabolic syndromes such as cardiovascular events, hyperlipidemia, and diabetes mellitus [15, 58], which may deteriorate the healthy status and indirectly lead to the decline of the OS rate of high VAT group. 

This study has several important limitations. First, the number of patients was small in our study, and female patients account for only 18.9% in the cohort; further studies with multi-center larger sample size are needed to confirm the results of this study. Second, this study was retrospective, patients who did not receive an abdominal CT scan within 3 months before LT were not included in the study, and this may have caused selection bias. Although PSM analysis was used to reduce the bias, the results may be affected by unconsidered factors. Third, due to the lack of donor and operation-related data, we are unable to analyze the risk factors of EAD comprehensively. Additionally, the follow-up time is short, and the direct effect of high VAT area on worse OS and graft survival are needed to be testified based on long-term follow-up in the future. 

In conclusion, LT recipients with a high amount of visceral fat were more likely to develop EAD. It also seems to have certain clinical value for predicting poor long-term prognosis of patients who underwent LT. More importantly, liver transplant candidates with high VAT area may be targets for timely therapeutic intervention to improve short- and long-term outcomes.

## Data Availability

The datasets used and/or analyzed during the current study are available from the corresponding author on reasonable request.

## Abbreviations

ALP: Alkaline phosphatase
ALT: Alanine aminotransferase
AST: Aspartate aminotransferase
BMI: Body mass index
CI: Confidence interval
CT: Computed tomography
EAD: Early allograft dysfunction
FFA: Free fatty acid
HBV: Hepatitis B virus
HCV: Hepatitis C virus
HR: Hazard ratio
HU: Hounsfield units
IL-6: Interleukin-6
IMAT: Intramuscular adipose tissue
INR: International normalized ratio
IQR: Interquartile range
IRI: Ischemic-reperfusion injury
LDH: Lactate dehydrogenase
LT: Liver transplantation
MELD: Model for end-stage liver disease
NLR: Neutrophil–lymphocyte ratio
OR: Odds ratio
OS: Overall survival
PLR: Platelet–lymphocyte ratio
PSM: Propensity-score matching
ROC: The receiver operating characteristic
SAT: Subcutaneous adipose tissue
SMA: Skeletal muscle area
SMD: The mean attenuation of skeletal muscle
TNF-ɑ: Tumor necrosis factor ɑ
VAT: Visceral adipose tissue
VSR: Visceral to subcutaneous adipose tissue area ratio
